# Amputation of the Thigh

**Published:** 1884-12

**Authors:** 


### AMPUTATION OF THE THIGH.

A CLINICAL LECTURE BY PROFESSOR W. F. WESTMORELAND, AT THE ATLANTA MEDICAL COLLEGE, OCT. 10, 1884.

Reported for the ATLANTA MEDICAL AND SURGICAL JOURNAL.

This case, gentlemen, is suffering from the effects of a burn, received fifty years ago. The patient says he is now fifty-seven years old, and that he received this burn when a small boy, and that he has never been entirely well since. At one time the ulcer came near healing, but for the past few years it has grown worse, until now he is frequently prostrated from the profuse hemorrhage that occurs from time to time. The ulcer, as you see, occupies the entire popliteal space of the left leg, extending some distance below and above the space. It is deepest just over the popliteal artery. So much of the tissues have been destroyed that you can see the pulsations of the popliteal artery. The danger to this patient now is, if left alone, that the vessels may become diseased, and rupture and death would result from the hemorrhage. This condition makes an operation necessary. We will make the operation at the junction of the middle and lower third, and in order to do this, we will have to make an irregular flap. We prefer the circular method, but in this case we cannot adopt it, in consequence of the extensive ulceration on the posterior surface. To make the circular operation we would have to amputate in the middle third, but by making our flap from the anterior surface, we can make the operation lower down. We do this for two reasons: First, it is less dangerous to the patient to make the operation in the lower third than higher up. Second, it is our duty always to leave as much stump as possible, in order that the patient may wear an artificial leg. In this case, however, it makes very little difference, for this patient is a life-time convict in the penitentiary, and the lessees will not be apt to furnish him with an artificial leg, any way. The patient has been very anæmic for some time, and in order that we may give him every chance of recovery, we make a bloodless operation. We do this by applying a rubber bandage from the toes to the upper part of the thigh. In this way we drive out all the blood, and the patient will lose comparatively none. After applying this bandage as high as necessary, we now surround the thigh with this rubber tubing, to prevent the return flow of blood after removal of the bandage. We introduce the knife, as you see, a little above the juncture of the middle and lower third of the thigh, and carry the incision downwards towards the knee, until we reach the patella, then we make the same incision on the under surface of the thigh making the two meet at the patella. On the posterior surface we make a circular cut until it reaches the two incisions already made. We now dissect back the skin, and with two strokes of the knife sever the muscles to the bone. Then with this retractor, the assistant will hold the soft parts out of the way of the saw, and we at once sever the bone.

You will see now that the limb has been removed, and there has not been hemorrhage enough to stain the knife. We will now secure the principal arteries, remove the rubber tubing, and then we will be able to secure any small branches that do not now show themselves. You will notice what seems to be a redundancy of flap, but when he revives and the muscles regain their contractility, there will be considerable reduction of flap, and then we will see that there is not too much. Do not make the mistake of leaving too little flap in any operation.

The dressing of this will consist of closing the wound with silver wire suture, with adhesive straps to support it, and carbolized water dressing for a few days.

